# Plasma Membrane Ca^2+^–ATPase in Rat and Human Odontoblasts Mediates Dentin Mineralization

**DOI:** 10.3390/biom11071010

**Published:** 2021-07-10

**Authors:** Maki Kimura, Hiroyuki Mochizuki, Ryouichi Satou, Miyu Iwasaki, Eitoyo Kokubu, Kyosuke Kono, Sachie Nomura, Takeshi Sakurai, Hidetaka Kuroda, Yoshiyuki Shibukawa

**Affiliations:** 1 Department of Physiology, Tokyo Dental College, 2-9-18, Kanda-Misaki-cho, Chiyoda-ku, Tokyo 101-0061, Japan; tsumuramaki@tdc.ac.jp (M.K.); h.motsu@yr.tnc.ne.jp (H.M.); kyosuke@pony.ocn.ne.jp (K.K.); snom0112@gmail.com (S.N.); ken5tuna@yahoo.co.jp (T.S.); kuroda@kdu.ac.jp (H.K.); 2 Department of Epidemiology and Public Health, Tokyo Dental College, Chiyodaku, Tokyo 101-0061, Japan; satouryouichi@tdc.ac.jp (R.S.); iwasakimiyu@tdc.ac.jp (M.I.); 3 Department of Microbiology, Tokyo Dental College, Chiyodaku, Tokyo 101-0061, Japan; kokubu@tdc.ac.jp; 4 Department of Dental Anesthesiology, Kanagawa Dental University, 1-23, Ogawacho, Kanagawa, Yokosuka-shi 238-8570, Japan

**Keywords:** dentinogenesis, odontoblasts, plasma membrane calcium-transporting ATPases, calcium signaling

## Abstract

Intracellular Ca^2+^ signaling engendered by Ca^2+^ influx and mobilization in odontoblasts is critical for dentinogenesis induced by multiple stimuli at the dentin surface. Increased Ca^2+^ is exported by the Na^+^–Ca^2+^ exchanger (NCX) and plasma membrane Ca^2+^–ATPase (PMCA) to maintain Ca^2+^ homeostasis. We previously demonstrated a functional coupling between Ca^2+^ extrusion by NCX and its influx through transient receptor potential channels in odontoblasts. Although the presence of PMCA in odontoblasts has been previously described, steady-state levels of mRNA-encoding PMCA subtypes, pharmacological properties, and other cellular functions remain unclear. Thus, we investigated PMCA mRNA levels and their contribution to mineralization under physiological conditions. We also examined the role of PMCA in the Ca^2+^ extrusion pathway during hypotonic and alkaline stimulation-induced increases in intracellular free Ca^2+^ concentration ([Ca^2+^]_i_). We performed RT-PCR and mineralization assays in human odontoblasts. [Ca^2+^]_i_ was measured using fura-2 fluorescence measurements in odontoblasts isolated from newborn Wistar rat incisor teeth and human odontoblasts. We detected mRNA encoding PMCA1–4 in human odontoblasts. The application of hypotonic or alkaline solutions transiently increased [Ca^2+^]_i_ in odontoblasts in both rat and human odontoblasts. The Ca^2+^ extrusion efficiency during the hypotonic or alkaline solution-induced [Ca^2+^]_i_ increase was decreased by PMCA inhibitors in both cell types. Alizarin red and von Kossa staining showed that PMCA inhibition suppressed mineralization. In addition, alkaline stimulation (not hypotonic stimulation) to human odontoblasts upregulated the mRNA levels of dentin matrix protein-1 (DMP-1) and dentin sialophosphoprotein (DSPP). The PMCA inhibitor did not affect DMP-1 or DSPP mRNA levels at pH 7.4–8.8 and under isotonic and hypotonic conditions, respectively. We also observed PMCA1 immunoreactivity using immunofluorescence analysis. These findings indicate that PMCA participates in maintaining [Ca^2+^]_i_ homeostasis in odontoblasts by Ca^2+^ extrusion following [Ca^2+^]_i_ elevation. In addition, PMCA participates in dentinogenesis by transporting Ca^2+^ to the mineralizing front (which is independent of non-collagenous dentin matrix protein secretion) under physiological and pathological conditions following mechanical stimulation by hydrodynamic force inside dentinal tubules, or direct alkaline stimulation by the application of high-pH dental materials.

## 1. Introduction

Odontoblasts play critical roles in the generation of dentinal sensitivity (known as the odontoblast hydrodynamic receptor model [1]) and dentin formation (dentinogenesis) in both physiological and pathological settings. We previously reported that odontoblasts are sensory receptor cells capable of detecting multiple stimuli applied to the dentin surface [1,2,3,4,5,6,7,8]. These stimuli at the surface are transformed into dentinal fluid movements, which elicit intracellular Ca^2+^ signaling by increasing the concentration of intracellular free Ca^2+^ ([Ca^2+^]_i_) through Ca^2+^ influx activated by mechanosensitive ion channels, transient receptor potential (TRP) channel subtypes, and Piezo1 channels [1,5]. This increase in [Ca^2+^]_i_ results in intercellular odontoblast–odontoblast and odontoblast–neuron signal communication mediated by ATP and glutamate, which are released from mechanically stimulated odontoblasts [1,4,8]. ATP release from odontoblast pannexin-1 channels induced by the [Ca^2+^]_i_ increase activates ionotropic adenosine triphosphate receptors (P2X_3_ receptors) in intradental Aδ neurons, which induces an action potential in Aδ neurons, generating dentinal sensitivity [5]. In addition, the increased intracellular Ca^2+^ is extruded extracellularly to the mineralizing front by the Na^+^–Ca^2+^ exchanger (NCX) in odontoblasts [1,2,3,9] to maintain [Ca^2+^]_i_ homeostasis. The data show that intracellular Ca^2+^ signaling participates in sensory signal transduction sequences that produce dentinal pain or promote dentinogenesis. Therefore, in odontoblasts, the regulation of [Ca^2+^]_i_ is important for regulating cellular function.

Intracellular Ca^2+^ concentrations are tightly controlled by multiple plasma membrane/intracellular Ca^2+^ transport proteins. Transport mechanisms are mediated by transmembrane Ca^2+^ influx and intracellular Ca^2+^ mobilization, as well as Ca^2+^ extrusion mechanisms. The [Ca^2+^]_i_ increase induced by Ca^2+^ influx from the extracellular space or Ca^2+^ release from Ca^2+^ stores subsides and returns to resting [Ca^2+^]_i_ via the sarco/endoplasmic reticulum Ca^2+^–ATPase (SERCA)-mediated uptake of Ca^2+^ into Ca^2+^ stores or Ca^2+^ extrusion to the extracellular space [10,11]. The extrusion of increased [Ca^2+^]_i_ is catalyzed by NCX or plasma membrane Ca^2+^–ATPase (PMCA) [12]. Thus, NCX and PMCA play key roles in the maintenance of cellular Ca^2+^ homeostasis. In a previous study, we found that NCX1 and NCX3 were highly expressed on the distal membrane of odontoblasts [9]. However, in non-excitable cells, PMCA is the major driver of Ca^2+^ efflux from the cytosol [13]. PMCA belongs to the family of P-type (subclass P2B) ATPases, which use the high energy produced by ATP hydrolysis to carry Ca^2+^ against membrane electrochemical gradients [13,14,15].

Odontoblasts have been shown to express PMCA in the distal cell membrane and its processes [16,17]. Lundgren and Linde (1988 and 1995) previously demonstrated ATP-dependent Ca^2+^ extrusion, most likely by Ca^2+^–ATPase, across the plasma membrane in odontoblasts [18,19]. In addition, PMCA epitopes have been reported to be present in odontoblasts [17]. These results suggest the involvement of the PMCA in odontoblasts in regulating the delivery of Ca^2+^ to the mineralizing front and dentinogenesis. However, the levels of mRNA-encoding PMCA subtypes and the pharmacological properties and cellular roles of PMCA in odontoblasts have yet to be determined. 

In the present study, to elucidate the role of PMCA in the maintenance of intracellular calcium levels in odontoblasts and mineralization during dentinogenesis, we assessed PMCA1–4 mRNA levels in human odontoblasts (HOB cells). In addition, changes in the mRNA levels of non-collagenous extracellular matrix proteins in cells exposed to various external conditions, with or without a PMCA inhibitor, were evaluated. We also examined the role of PMCA during dentinogenesis using mineralizing assays, and investigated PMCA1 immunoreactivity using immunofluorescence analysis in HOB cells. Finally, to clarify the involvement of PMCA during Ca^2+^ extrusion, we measured Ca^2+^ extrusion efficiencies during hypotonic or high-pH stimulation-induced Ca^2+^ mobilization in acutely isolated rat and human odontoblasts.

## 2. Materials and Methods

### 2.1. Ethical Approval

All animals (*N* = 25 in total) were treated in accordance with the Guiding Principles for the Care and Use of Animals in the field of physiological sciences, which were approved by the Council of the Physiological Society of Japan and the American Physiological Society. All animal experiments were carried out in accordance with the guidelines established by the National Institutes of Health regarding the care and use of animals for experimental procedures. The experiments followed the United Kingdom Animal (Scientific Procedures) Act, 1986. All experimental protocols were approved by the Ethics Committee of Tokyo Dental College (numbers 300301, 190301, and 200301).

### 2.2. Odontoblast Cell Culture 

The human odontoblast cell line (HOB cell) was obtained from a healthy third molar and immortalized by transfection with the human telomerase transcriptase gene [6,20,21]. The resulting cells showed mRNA expression of dentin sialophosphoprotein (DSPP), type 1 collagen, alkaline phosphatase, and bone sialoprotein, and exhibited nodule formation by Alizarin red staining in the mineralizing medium [20]. HOB cells were cultured in basal medium containing alpha-minimum essential medium, 10% fetal bovine serum (FBS), 1% penicillin/streptomycin (Life Technologies Japan, Tokyo, Japan), and 1% amphotericin B (Sigma-Aldrich, St Louis, MO, USA) at 37 °C in a 5% CO_2_ incubator.

### 2.3. Acute Isolation of Rat Odontoblasts from Dental Pulp Slices 

Dental pulp slices were obtained from newborn Wistar rats (5−8 days old) using a previously described method [1,2,6,22,23,24]. The animals were housed with food and water available ad libitum. Briefly, the mandible was dissected under isoflurane (3%) and pentobarbital sodium (25 mg/kg, i.p.) anesthesia. The hemimandible was embedded in alginate impression material and sliced transversely through the incisor at a 500 µm thickness using a standard vibrating tissue slicer (ZERO 1; Dosaka EM, Kyoto, Japan). A section of the mandible was sliced to the level at which the dentin and enamel were directly visible between the bone tissue and the dental pulp. Mandible sections in which the dentin layer was thin and the enamel and dentin were clearly distinguishable by microscopy were selected to avoid cellular damage to odontoblasts. The surrounding impression material, enamel, bone tissue, and dentin were removed from the mandible section under a stereomicroscope. The remaining dental pulp slices were used in further experiments. Pulp slices were treated with standard Krebs solution supplemented with 0.17% collagenase and 0.03% trypsin (30 min at 37 °C). For [Ca^2+^]_i_ measurement, enzymatically treated dental pulp slices were plated onto a culture dish, soaked in alpha-minimum essential medium containing 5% horse serum and 10% fetal bovine serum (Life Technologies Japan), and maintained at 37 °C in a 5% CO_2_ incubator. [Ca^2+^]_i_ in odontoblasts located on the periphery of the primary cultured dental pulp slices was measured within 24 h of isolation. In a previous study, cells isolated using the same protocol were validated as odontoblasts with positive immunofluorescence for odontoblast marker proteins, dentin matrix protein-1 (DMP-1), dentin sialoprotein, and nestin within 24 h of isolation [2].

### 2.4. RT-PCR

For real-time RT-PCR analysis, HOB cells were cultured under physiological or high-pH conditions, as well as isotonic or hypotonic conditions, for 3 days with or without 5(6)-carboxyeosin (CE) (10 µM). To obtain HOB cells under high-pH conditions, culture plates with basal medium were maintained in an incubator (37 °C) without CO_2_ for 12 h per day (21:00–09:00) for 3 days. We also examined the effects of isotonic or hypotonic conditions on the changes in levels of the mRNA of interest. For the isotonic medium, we prepared modified basal medium by reducing NaCl to 28.7 mM and adding 150 mM mannitol (300 mOsm/L) (Functional Peptides. Co., Higashine, Japan). To prepare the hypotonic medium, we modified the medium by reducing NaCl to 28.7 mM and adding 50 mM mannitol (200 mOsm/L) (Functional Peptides. Co.). HOB cells were also cultured in each medium for 3 days, with or without CE (10 µM). 

Total RNA from HOB cells was extracted using the modified acid guanidium–phenol–chloroform method. The purity and concentration of total RNA was determined using a NanoDrop ND-2000 instrument (Thermo Fisher Scientific, Waltham, MA, USA), and 50 ng/μL of total RNA was used for the RT-PCR analysis. In addition, RNA integrity and the RNA integrity number (RIN) were determined using Agilent 4200 TapeStation (Agilent Technologies, Inc., Santa Clara, CA, USA). The 28S:18S rRNA ratio was 1.80 ± 0.24, and the RIN was 9.66 ± 0.25 (47 samples). Reverse transcription, complementary DNA amplification, and PCR were performed using a One-Step SYBR Primescript RT-PCR Kit with Thermal Cycler Dice for semi-quantitative real-time RT-PCR (TaKaRa-Bio, Shiga, Japan). Primer sets and PCR conditions are listed in Table 1. Real-time RT-PCR data were quantified using the comparative threshold (2^−ΔΔCt^) method [25,26]. Levels of the mRNA of interest were normalized to β-actin levels. Mean Ct values were obtained for each primer set. Changes in Ct were calculated as the difference between the average Ct for the target gene and β-actin as the control for the total starting RNA quantity. Fold changes in mRNA levels relative to β-actin mRNA levels were assessed using the ΔΔCt method.

### 2.5. Fluorescence Measurement of [Ca^2+^]_i_

Odontoblasts in dental pulp slices and HOB cells were loaded with 10 μM fura-2-acetoxymethyl ester (Dojindo Laboratories, Kumamoto, Japan) [27] and 0.1% (*w/v*) pluronic acid F-127 (Life Technologies, Japan) in Krebs solution at 37 °C. After 30 min of loading fura-2, they were rinsed with fresh Krebs solution. Fura-2-loaded odontoblasts were imaged by fluorescence microscopy (IX73; Olympus, Tokyo, Japan) with an excitation wavelength selector, HCImage software, and an intensified charge-coupled device camera system (Hamamatsu Photonics, Shizuoka, Japan). Fura-2 fluorescence was recorded at 510 nm in response to excitation wavelengths of 380 nm (F380) and 340 nm (F340). The [Ca^2+^]_i_ was determined using the fluorescence ratio (R_F340/F380_) of F340 to F380, which is represented as F/F_0_ units; the R_F340/F380_ value (F) was normalized to the resting value (F_0_). The F/F_0_ baseline was arbitrarily set at 1.0. All experiments were carried out at room temperature (25 ± 1.0 °C). Hypotonic, isotonic, high-pH, and Krebs solutions and those containing PMCA inhibitors were applied by superfusion using a rapid gravity-fed perfusion system (ValveLink8.2 Controller; AutoMate Scientific, Berkeley, CA, USA). It is worth noting that these solutions included 2.5 mM extracellular Ca^2+^. At the start of the experiment using caloxin 1b1, the odontoblasts in the recording chamber were perfused with a standard extracellular solution or isotonic solution. By changing the standard extracellular solution or isotonic solution to high-pH or hypotonic solution, respectively, with caloxin 1b1 (see Figure legends for details), followed by changing the solution into a standard or isotonic solution, we analyzed hypotonic- or high-pH-mediated increases and extrusion of [Ca^2+^]_i_. For the experiments using CE, the odontoblasts were perfused by standard extracellular or isotonic solution with CE, and the solution was changed to high-pH or hypotonic solution with CE, followed by changing the solution to a standard extracellular or isotonic solution with CE (see Figure legends for details). For the control, the cells were perfused by standard extracellular or isotonic solution, and the solution was changed to high-pH or hypotonic solution, followed by changing the solution to a standard extracellular or isotonic solution.

### 2.6. Mineralization Assay

HOB cells were grown to full confluency in basal medium and then transferred to mineralization medium (10 mM β-glycerophosphate and 100 μg/mL ascorbic acid in basal medium) for growth at 37 °C in 5% CO_2_. To determine the effects of PMCA activity on mineralization, HOB cells were cultured in mineralization medium without (as control) or with PMCA inhibitors CE (10 µM) or caloxin 1b1 (100 µM) for 28 days. During the 28-day culture period, mineralization media with or without PMCA inhibitors was changed twice a week [6]. To detect the deposition of calcium and calcium phosphate [6,28], cells were subjected to Alizarin red and von Kossa staining, and the mineralizing efficiencies were measured using ImageJ software (NIH, Maryland, USA). Images were obtained with a digital camera (Sony, Tokyo, Japan), converted to 8-bit, and converted to reversed grayscale. Regions of interest (ROIs) were then determined for each whole well to measure the mean luminance intensities of the total pixel numbers (I) of the ROI. Mineralizing efficiencies were normalized and represented as I/I_0_ units; the intensities (I) of Alizarin red and von Kossa staining were normalized to the mean intensity values of areas without cells (I_0_).

### 2.7. Solutions and Reagents

Krebs solution containing 136 mM NaCl, 5 mM KCl, 2.5 mM CaCl_2_, 0.5 mM MgCl_2_, 10 mM HEPES, 10 mM glucose, and 12 mM NaHCO_3_ (pH 7.4, Tris) was used as a standard extracellular solution. The isotonic solution was comprised of 36 mM NaCl, 200 mM mannitol, 5 mM KCl, 2.5 mM CaCl_2_, 0.5 mM MgCl_2_, 10 mM HEPES, 10 mM glucose, and 12 mM NaHCO_3_ (pH 7.4, Tris). To induce membrane stretching [3,29], a hypotonic solution (200 mOsm/L) was prepared by decreasing the concentration of mannitol to 64 mM. For the high-pH (pH 8) extracellular solution, 12 mM NaHCO_3_ in Krebs solution was changed to 8 mM (pH 8) NaOH [6,7]; this change had no effect on the extracellular free Ca^2+^ concentration in the test solution. Caloxin 1b1 was obtained from Karebay Biochem, Inc. (Monmouth Junction, NJ, USA). CE was obtained from Abcam (Cambridge, UK). All other reagents were purchased from Sigma-Aldrich. A stock solution of caloxin 1b1 was prepared in 10% ethanol. A CE stock solution was prepared in dimethyl sulfoxide. Stock solutions were diluted to the appropriate concentrations using the Krebs, isotonic, hypotonic, high-pH solution, or medium before use. The concentrations of the PMCA inhibitors used in this study were determined by Groten et al. (2016) and Chen et al. (2014) for CE, while those for caloxin 1b1 were determined according to Pande et al. (2006 and 2008) [11,30,31,32].

### 2.8. Immunostaining

HOB cells were cultured in 8-well glass chambers (AGC Techno Glass Co.,Ltd., Shizuoka, Japan) for 1 day. Cells were fixed with 4% paraformaldehyde (FUJIFILM Wako Pure Chemical Co., Osaka, Japan) and washed with 1× PBS (Thermo Fisher Scientific K.K., Tokyo, Japan). After 10 min of incubation with blocking buffer (Nacalai Tesque, Kyoto, Japan) at room temperature, mouse monoclonal anti-PMCA1 (Santa Cruz Biotechnology, Dallas, Texas, USA; sc-398413, F-10, 1:200) was applied for 6 h to detect human PMCA1. Secondary antibody (Alexa Fluor^®^ 555 donkey anti-mouse; Thermo Fisher Scientific K.K.) was then applied for 1 h. Stained samples were mounted in mounting medium containing 4,6-diamidino- 2-phenylindole (Abcam, Cambridge, UK). Immunostained samples were analyzed and observed using a fluorescence microscope (BZ9000; KEYENCE Co., Osaka, Japan). For negative control, the cells were incubated with nonimmune antibody diluted to equivalent concentrations to that of the primary antibody (data not shown).

### 2.9. Statistics and Offline Analysis

Data are presented as means ± standard errors (SE) of the mean of *N* observations, where *N* represents the number of independent experiments. Parametric statistical significance was determined using one-way ANOVA with Tukey’s post-hoc test to analyze detected PMCA mRNA levels in HOB cells. Non-parametric statistical significance was determined using the Mann–Whitney test or Kruskal–Wallis test with Dunn’s post-hoc test. Statistical significance was set at *P* < 0.05. Statistical analyses were conducted using GraphPad Prism 7.0 (GraphPad Software, La Jolla, CA, USA). 

## 3. Results 

### 3.1. Measurement of PMCA1–4 mRNA Levels in Human Odontoblasts

**Figure 1:**
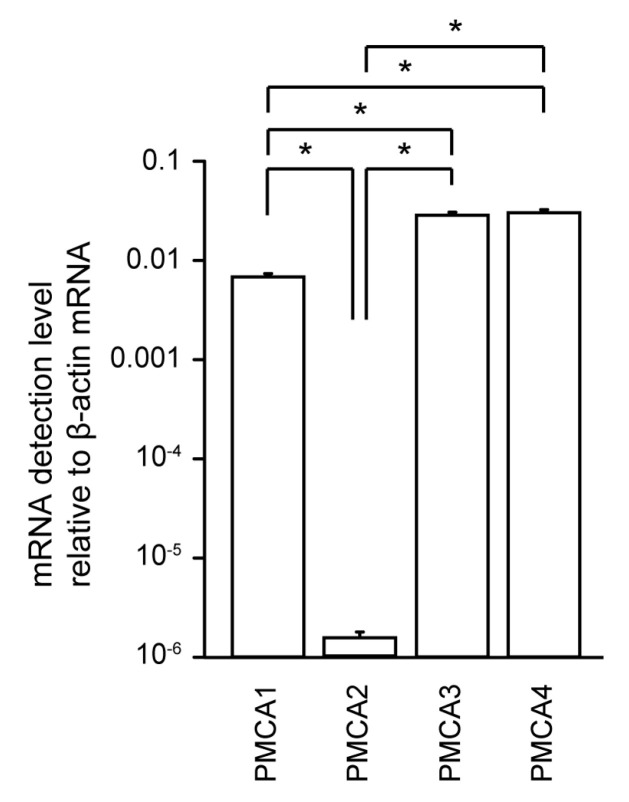
mRNA levels of plasma membrane Ca^2+^–ATPase (PMCA)1–4 in human odontoblast cell lines (HBO cells). Real-time RT-PCR was used to quantify the detection level of PCMA1–4 mRNAs by measuring the increase in fluorescence elicited by the binding of SYBR green dye to double-stranded DNA. Data were analyzed by the 2^−[ΔΔCt]^ method, with β-actin as an internal control (which was positive in all samples). Each bar denotes the mean ± SE of 7 experiments. Expression levels were normalized to β-actin mRNA levels. Statistically significant differences between columns (shown by solid lines) are marked with asterisks. * *P* < 0.05.

Using real-time RT-PCR analysis, we robustly detected mRNAs encoding PMCA1, PMCA3, and PMCA4 in HOB cells, but the detected level of PMCA2 mRNA was significantly lower than that of its paralogs (Figure 1). 

### 3.2. Extrusion of Ca^2+^ by PMCA Following Hypotonic or High-pH Stimulation in Acutely Isolated Rat Odontoblasts and HOB Cells

**Figure 2:**
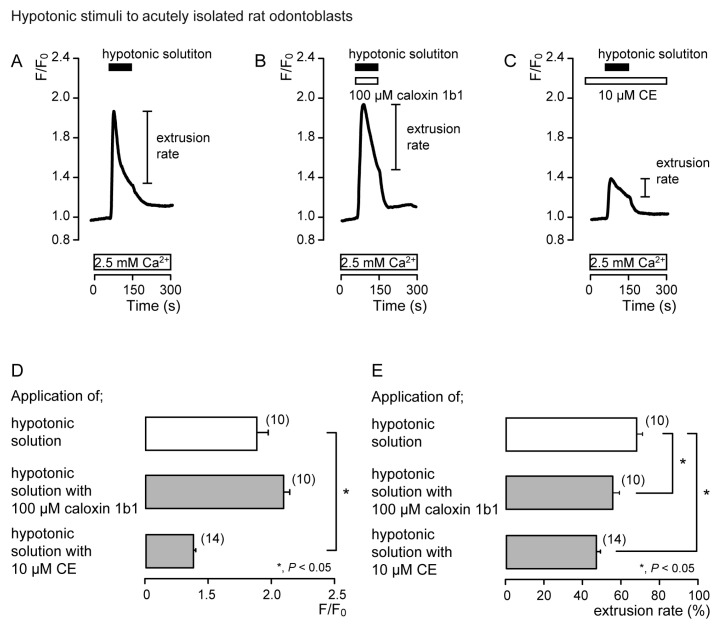
PMCA mediates Ca^2+^ extrusion following hypotonic stimuli in acutely isolated rat odontoblasts. (**A**–**C**) Representative traces of intracellular free Ca^2+^ concentration ([Ca^2+^]_i_) following hypotonic stimuli (black boxes at tops of graphs) in the absence of 100 μM caloxin 1b1 and 10 μM 5(6)-carboxyeosin (CE) (**A**), with 100 μM caloxin 1b1 (white box at the top) (**B**), or with 10 μM CE (white box at the top) (**C**), in the presence of extracellular Ca^2+^ (white boxes at bottom in A to C). (**D**) Peak values of [Ca^2+^]_i_ during hypotonic stimuli without 100 μM caloxin 1b1 and 10 μM CE (open column; 1.88 ± 0.08 F/F_0_ units), with 100 μM caloxin 1b1 (upper gray column; 2.08 ± 0.05 F/F_0_ units), and with 10 μM CE (lower gray column; 1.37 ± 0.03 F/F_0_ units). Each column denotes the mean ± SE from 10, 10, and 14 independent experiments, respectively. (**E**) Ca^2+^ extrusion rates during hypotonic stimulation in the absence of 100 μM caloxin 1b1 and 10 μM CE (open column; 68.2 ± 2.8%), with 100 μM caloxin 1b1 (upper gray column; 55.8 ± 3.2%), and with 10 μM CE (lower gray column; 46.3 ± 2.4%). Each column denotes the mean ± SE from 10, 10, and 14 independent experiments, respectively. Statistically significant differences (in (**D**,**E**)) between columns (shown by solid lines) are indicated by asterisks. * *P* < 0.05.

**Figure 3:**
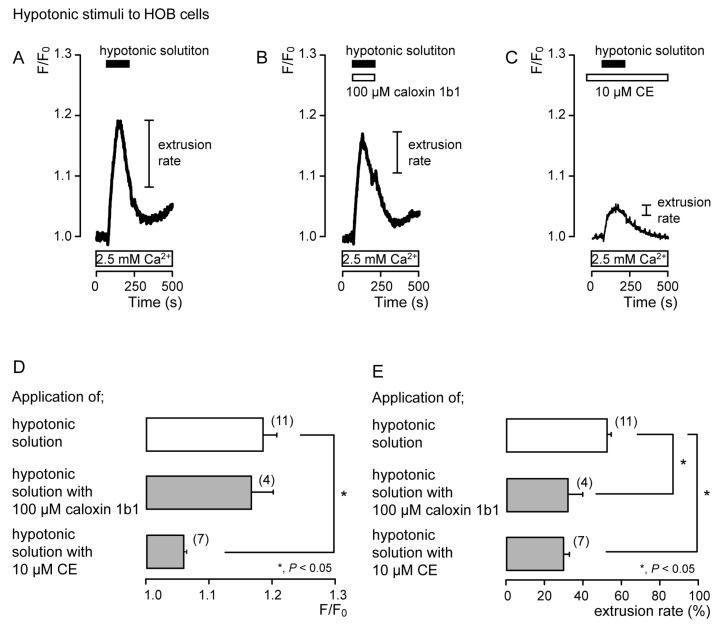
PMCA mediates Ca^2+^ extrusion following hypotonic stimuli in HOB cells. (**A**–**C**) Representative traces of [Ca^2+^]_i_ following hypotonic stimuli (black boxes at tops of graphs) in the absence of 100 μM caloxin 1b1 and 10 μM CE (**A**), with 100 μM caloxin 1b1 (white box at the top) (**B**), or with 10 μM CE (white box at the top) (**C**), in the presence of extracellular Ca^2+^ (white boxes at bottom in A to C). (**D**) Peak values of [Ca^2+^]_i_ during hypotonic stimuli without 100 μM caloxin 1b1 and 10 μM CE (open column; 1.19 ± 0.02 F/F_0_ units), with 100 μM caloxin 1b1 (upper gray column; 1.17 ± 0.03 F/F_0_ units), and with 10 μM CE (lower gray column; 1.06 ± 0.00 F/F_0_ units). Each column denotes the mean ± SE from 11, 4, and 7 independent experiments, respectively. (**E**) Ca^2+^ extrusion rates during hypotonic stimulation in the absence of 100 μM caloxin 1b1 and 10 μM CE (open column; 53.4 ± 2.2%), with 100 μM caloxin 1b1 (upper gray column; 32.4 ± 7.3%), and with 10 μM CE (lower gray column; 29.7 ± 3.0%). Each column denotes the mean ± SE from 11, 4, and 7 independent experiments, respectively. Statistically significant differences (in (**D**,**E**)) between columns (shown by solid lines) are indicated by asterisks. * *P* < 0.05.

**Figure 4:**
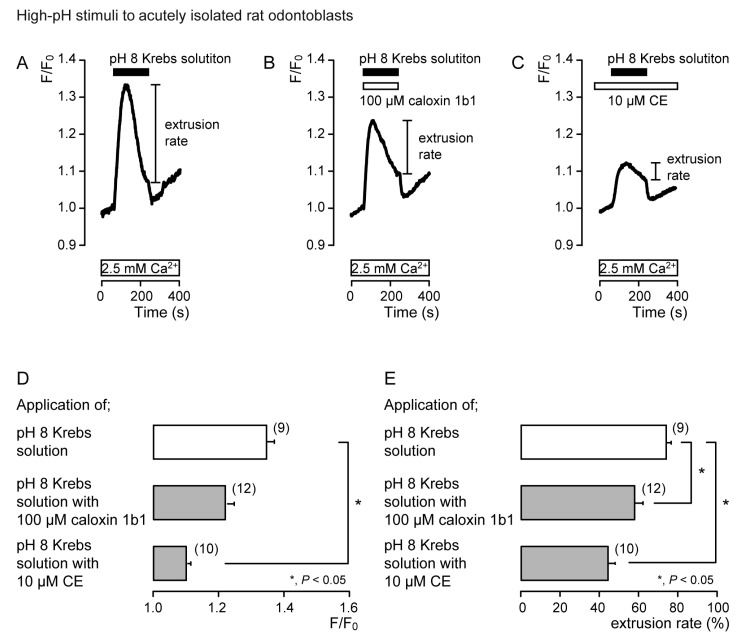
PMCA mediates Ca^2+^ extrusion following high-pH stimuli in acutely isolated rat odontoblasts. (**A**–**C**) Representative traces for pH 8 (black boxes)-induced [Ca^2+^]_i_ increases, in the absence of 100 μM caloxin 1b1 and 10 μM CE (**A**), with 100 μM caloxin 1b1 (white box at the top) (**B**), or with 10 μM CE (white box at the top) (**C**), in the presence of extracellular Ca^2+^ (white boxes at bottom in A to C). (**D**) Peak values of [Ca^2+^]_i_ during high-pH stimulation in the absence of 100 μM caloxin 1b1 and 10 μM CE (open column; 1.35 ± 0.02 F/F_0_ units), with 100 μM caloxin 1b1 (upper gray column; 1.23 ± 0.02 F/F_0_ units), and with 10 μM CE (lower gray column; 1.11 ± 0.01 F/F_0_ units). Each column denotes the mean ± SE from 9, 12, and 10 independent experiments, respectively. (**E**) Ca^2+^ extrusion rates during high-pH stimulation in the absence of 100 μM caloxin 1b1 and 10 μM CE (open column; 74.2 ± 2.5%), with 100 μM caloxin 1b1 (upper gray column; 58.0 ± 4.2%), and with 10 μM CE (lower gray column; 44.0 ± 3.9%). Each column denotes the mean ± SE from 9, 12, and 10 independent experiments, respectively. Statistically significant differences (in (**D**,**E**)) between columns (shown by solid lines) are indicated by asterisks. * *P* < 0.05.

**Figure 5:**
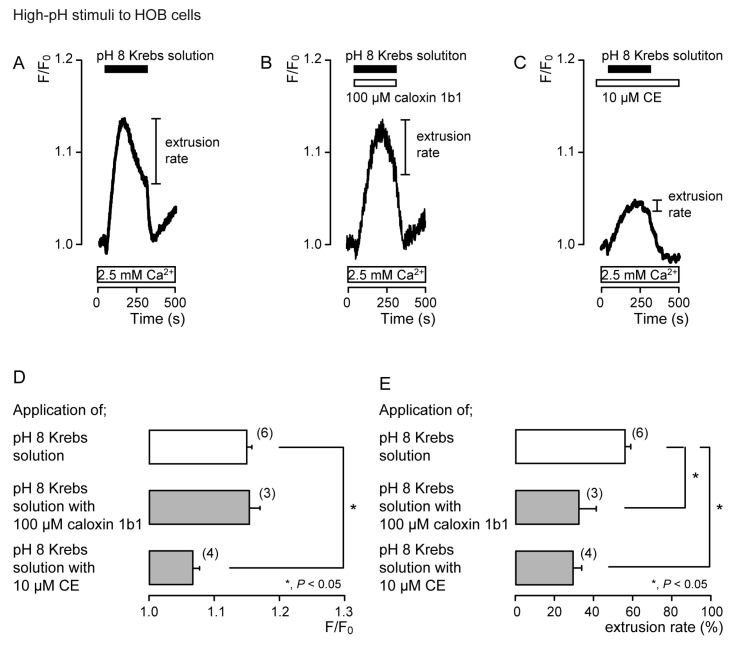
PMCA mediates Ca^2+^ extrusion following high-pH stimuli in HOB cells. (**A**–**C**) Representative traces for pH 8 (black boxes)-induced [Ca^2+^]_i_ increases, in the absence of 100 μM caloxin 1b1 and 10 μM CE (**A**), with 100 μM caloxin 1b1 (white box at the top) (**B**), or with 10 μM CE (white box at the top) (**C**), in the presence of extracellular Ca^2+^ (white boxes at bottom in A to C). (**D**) Peak values of [Ca^2+^]_i_ during high-pH stimulation in the absence of 100 μM caloxin 1b1 and 10 μM CE (open column; 1.15 ± 0.01 F/F_0_ units), with 100 μM caloxin 1b1 (upper gray column; 1.15 ± 0.02 F/F_0_ units), and with 10 μM CE (lower gray column; 1.07 ± 0.01 F/F_0_ units). Each column denotes the mean ± SE from 6, 3, and 4 independent experiments, respectively. (**E**) Ca^2+^ extrusion rates during high-pH stimulation in the absence of 100 μM caloxin 1b1 and 10 μM CE (open column; 55.6 ± 3.0%), with 100 μM caloxin 1b1 (upper gray column; 32.4 ± 8.5%), and with 10 μM CE (lower gray column; 29.0 ± 4.4%). Each column denotes the mean ± SE from 6, 3, and 4 independent experiments, respectively. Statistically significant differences (in (**D**,**E**)) between columns (shown by solid lines) are indicated by asterisks. * *P* < 0.05.

To demonstrate the contribution of PMCA to Ca^2+^ extrusion following mechanosensitive- or high-pH sensitive-[Ca^2+^]_i_ increases, we measured [Ca^2+^]_i_ and analyzed the extrusion efficiencies following the application of hypotonic or high-pH (pH 8) solutions with or without non-selective PMCA inhibitors (10 μM CE [11,31,33] or 100 μM caloxin 1b1 [15,30,32]) in acutely isolated rat odontoblasts (Figures 2 and 4) and HOB cells (Figures 3 and 5). The Ca^2+^ extrusion efficiency was determined by the extrusion rate, calculated as:

Extrusion rate (%) = (F/F_0peak_ − F/F_0 at termination of stimulation_)/(F/F_0peak_ − F/F_0_) (1)

where F/F_0_ is the baseline, F/F_0peak_ is the peak value of F/F_0_ upon stimulation, and F/F_0 at termination of stimulation_ is the value at the termination of stimulation. The F/F_0_ baseline ratio was normalized to 1.

In the presence of extracellular Ca^2+^, hypotonic stimulation transiently increased [Ca^2+^]_i_ in both the absence (Figure 2A and Figure 3A) and the presence of caloxin 1b1 (Figure 2B and Figure 3B) and CE (Figure 2C and Figure 3C) in acutely isolated rat odontoblasts (Figure 2) and HOB cells (Figure 3). In the presence of 10 μM CE (Figure 2C and Figure 3C), the peak values of hypotonic stimulation-induced [Ca^2+^]_i_ increases were significantly lower than those in the absence of CE and caloxin 1b1 in both cultured and isolated cells (Figure 2D and Figure 3D). These [Ca^2+^]_i_ increases decayed during the administration of hypotonic solution in both cells (Figure 2A–C and Figure 3A–C). In the presence of 100 µM caloxin 1b1 or 10 µM CE, the extrusion rate in [Ca^2+^]_i_ during hypotonic stimulation decreased significantly relative to that in the absence of PMCA inhibitors in both cultured and isolated cells (Figure 2E and Figure 3E). 

In the presence of extracellular Ca^2+^, the application of Krebs solution (pH 8) to acutely isolated rat odontoblasts (Figure 4) and HOB cells (Figure 5) transiently increased [Ca^2+^]_i_ in the presence of caloxin 1b1 (Figure 4B and Figure 5B) and CE (Figure 4C and Figure 5C), as well as in the control cells in their absence (Figure 4A and Figure 5A). The [Ca^2+^]_i_ increase with the addition of Krebs solution (pH 8) in the presence of 10 μM CE (Figure 4C and Figure 5C) was significantly lower than that in the absence of CE and caloxin 1b1 in both cultured and isolated cells (Figure 4D and Figure 5D). These [Ca^2+^]_i_ increases showed a decay in the increase during high-pH stimulation in both cells. The application of 100 µM caloxin 1b1 (Figure 4B and Figure 5B) or 10 µM CE (Figure 4C and Figure 5C) significantly decreased the extrusion rates of the high-pH-induced [Ca^2+^]_i_ increase in both cell types (Figure 4E and Figure 5E).

### 3.3. PMCA Mediates Mineralization by Odontoblasts

**Figure 6:**
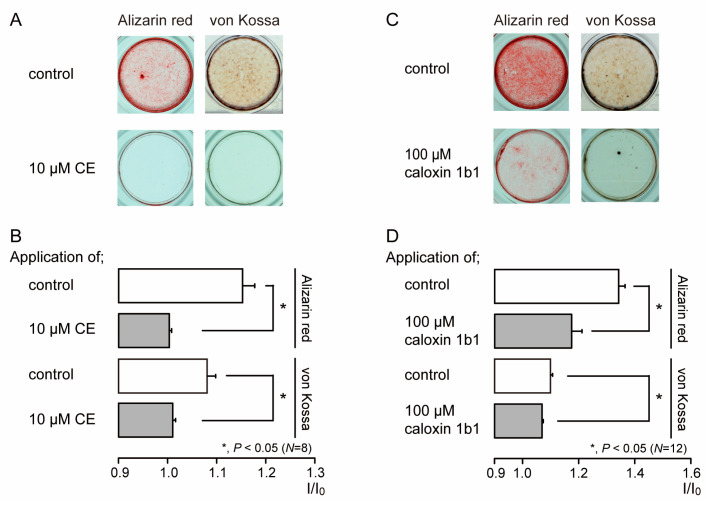
PMCA inhibitors decrease mineralization levels. (**A**,**C**) HOB cells were cultured for 28 days in mineralization medium with PMCA inhibitors (each lower column), 10 μM CE (**A**), or 100 μM caloxin 1b1 (**C**), or without them (each upper column in (**A**,**C**)) at pH 7.4. Alizarin red (left columns; red indicative of calcium deposition) and von Kossa (right columns; black indicative of phosphate and calcium deposition) staining. (**B**,**D**) Mineralization levels with (gray columns) and without (open columns) PCMA inhibitors 10 μM CE (**B**) or 100 μM caloxin 1b1 (**D**), assessed by Alizarin red (upper columns), and von Kossa (lower columns) staining. Each column in (**B**,**D**) denotes the mean ± SE from 8 and 12 experiments, respectively. The mineralization levels in the absence of CE (as controls in (**B**)) were 1.15 ± 0.03 I/I_0_ with Alizarin red staining and 1.08 ± 0.02 I/I_0_ units with von Kossa staining. The mineralization levels with CE (gray bars in (**B**)) were 1.00 ± 0.01 I/I_0_ units with Alizarin red staining and 1.01 ± 0.01 I/I_0_ units with von Kossa staining. The mineralization levels without caloxin 1b1 (as controls in (**D**)) were 1.34 ± 0.02 I/I_0_ units with Alizarin red staining and 1.11 ± 0.01 I/I_0_ units with von Kossa staining. The mineralization levels with caloxin 1b1 (gray bars in (**D**)) were 1.18 ± 0.04 I/I_0_ units with Alizarin red staining and 1.07 ± 0.01 I/I_0_ with von Kossa staining. Statistically significant differences (in (**B**,**D**)) between columns (shown by solid lines) are indicated by asterisks. * *P* < 0.05.

We investigated the effects of PMCA activity on mineralization induced by human odontoblasts. Alizarin red (left) and von Kossa (right) staining (Figure 6A,C) were determined to be indicative of the mineralization levels based on the staining intensity (see Materials and Methods) represented as I/I_0_ units; the intensities (I) of both stains were normalized to the mean intensities of adjacent areas without cells (I_0_). We compared the mineralization levels in HOB cells cultured in mineralization medium for 28 days in the presence or absence of PMCA inhibitors. The application of 10 µM CE (Figure 6A,B) or 100 µM caloxin 1b1 (Figure 6C,D) to mineralization media resulted in decreased mineralization levels compared to those without CE or caloxin 1b1 (controls). 

### 3.4. Effects of PMCA Inhibitor on Detected mRNA Levels of Non-Collagenous Extracellular Matrix Proteins as Odontoblast Markers

**Figure 7:**
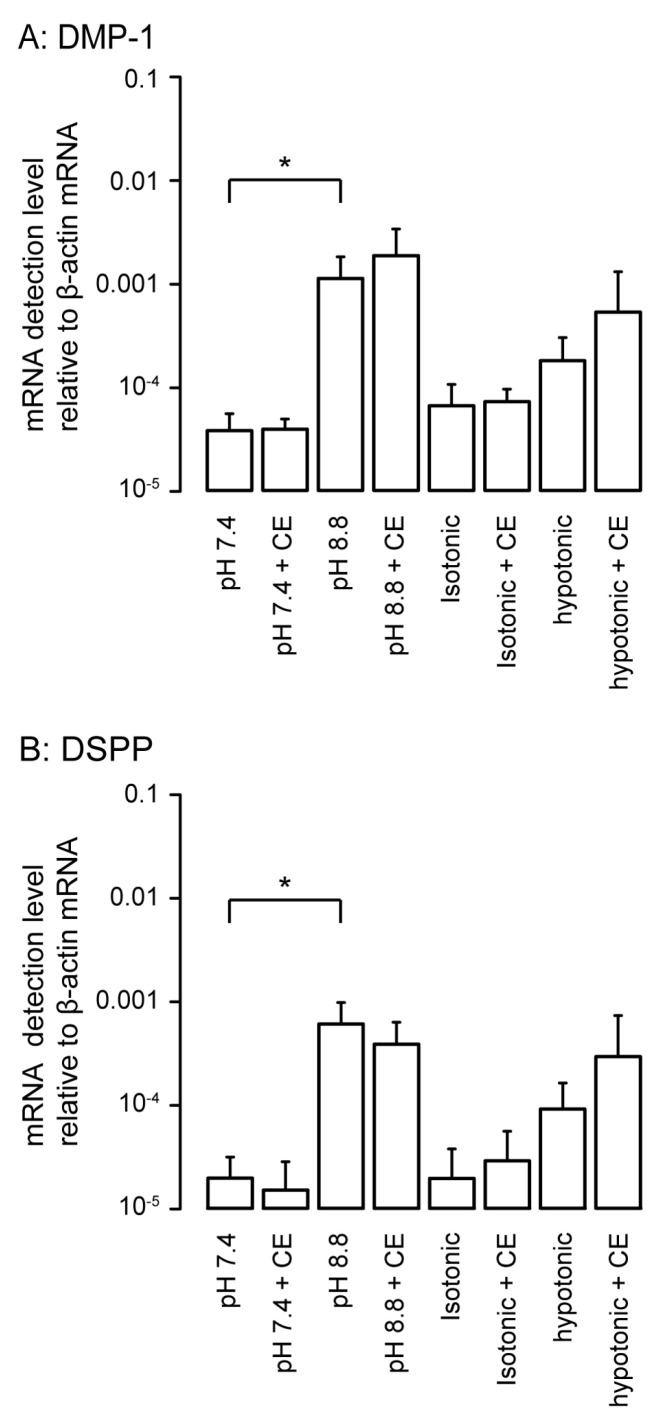
The changes in mRNA level of dentin matrix protein-1 (DMP-1) (**A**) and dentin sialophosphoprotein (DSPP) (**B**) as markers specific for odontoblasts in response to high-pH or hypotonic stimulation with or without CE in HOB cells. Real-time RT-PCR was used to quantify mRNA levels by measuring the increase in fluorescence elicited by the binding of SYBR green dye to double-stranded DNA. Data were analyzed by the 2^−[ΔΔCt]^ method, with β-actin as an internal control (which was positive in all samples). Each bar denotes the mean ± SE of 5 experiments under physiological or high-pH condition and 3 experiments for isotonic or hypotonic conditions. The levels of mRNA were normalized to β-actin mRNA levels. Statistically significant differences between columns (Shown by solid lines) are marked with asterisks. * *P* < 0.05.

We analyzed the changes in the mRNA levels of non-collagenous extracellular matrix proteins, such as odontoblast markers, dentin matrix protein-1 (DMP-1), and dentin sialophosphoprotein (DSPP), following 3 days of HOB cell culture under physiological or high-pH conditions as well as isotonic or hypotonic conditions with or without 10 µM CE. High-pH stimulation significantly increased the mRNA levels of DMP-1 (Figure 7A) and DSPP (Figure 7B) compared to stimulation at pH 7.4. We did not observe any significant differences in the mRNA levels between hypotonic and isotonic stimulation; however, hypotonic stimulation tended to increase the mRNA levels of DMP-1 and DSPP compared to those under isotonic conditions. The PMCA inhibitor, CE, did not have any significant effects on their mRNA levels in the normal and high-pH as well as isotonic and hypotonic extracellular conditions in odontoblasts. 

### 3.5. Immunofluoresence Analysis of PMCA1 in HOB Cells

**Figure 8:**
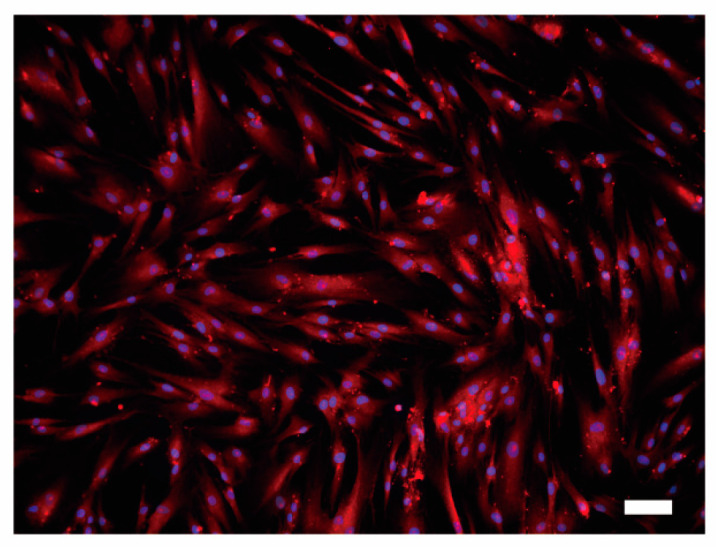
Immunofluorescence analysis of PMCA1 protein in HOB cells. HOB cells were positive for PMCA1 immunoreactivity (red). Nuclei are shown in blue. Scale bar: 100 μm. No fluorescence was detected in the negative control (not shown).

We further observed the presence of the ubiquitously expressed PMCA1 [11,14] protein in HOB cells by immunofluorescence staining. Distinct PMCA1 immunoreactivity on HOB cells was observed throughout the cell membrane (Figure 8).

## 4. Discussion

We have demonstrated the pharmacological properties of PMCAs and their roles in the cellular functions of odontoblasts. We have also shown the detection of mRNAs encoding PMCAs and PMCA1 immunoreactivity in human odontoblasts. In mammals, four PMCAs are numbered (1–4) [14]. PMCA1 and PMCA4 are expressed ubiquitously, whereas PMCA2 and PMCA3 have tissue-specific distributions [11,14]. It has been reported that the stereocilia of hair cells express PMCA2 at high levels, and PMCA2 defects are linked to profound hearing impairment/loss in humans [12]. The ablation of the *PMCA4* gene causes male infertility [12]. Although the ablation of the *PMCA1* gene results in early embryonic lethality (highlighting the essential role of PMCA1 in development, organogenesis, and housekeeping function [12]), the deletion of *PMCA1* in the intestine is associated with reduced bone mineralization [34]. In addition, osteoblasts and ameloblasts, which form hard tissues and play roles in bone mineralization and amelogenesis, respectively, also express PMCA [16,17,35,36]. In ameloblasts, PMCA activity increases gradually during developmental processes, suggesting that PMCA in ameloblasts is associated with amelogenesis and the maturation of ameloblasts [16,36]. During odontoblast differentiation, immunohistochemical analysis using monoclonal antibodies against erythrocyte PMCA revealed a lack of immunoreactivity in the pre-odontoblasts located at the surface of the dental papilla, while gradient increases in the immunoreaction intensity were observed in the period when the odontoblast became fully differentiated and in parallel to the progression of mineralization of dentin [17]. These previous results [16,17] are in line with our results showing the functional expression of PMCA in odontoblasts. In addition, we detected mRNAs encoding PMCA1, PMCA3, and PMCA4, while the level of detectable PMCA2 mRNA was significantly lower than those of its paralogs. Notably, it is well documented that mRNA levels of ion transporters and channels do not perfectly correlate with their protein levels for reasons including microRNA regulation and post-transcriptional degradation. Although we have observed PMCA1 immunoreactivity in human odontoblasts, further studies to reveal detailed protein levels and cellular localization patterns for PMCA subtypes in odontoblasts, and to reveal changes in levels/localization during odontoblast differentiation, odontogenesis, and dentinogenesis, are of immediate interest.

In the present study, we also found that intracellular Ca^2+^ increased by hypotonic or high-pH stimulation was extruded into the extracellular space by PMCA activity. PMCA generally has a high affinity for Ca^2+^ (*K_d_* ~0.2 µM) but a lower capacity for Ca^2+^ transport than NCX [10,13,37]. This allows for the maintenance of low [Ca^2+^]_i_ in the resting state [13,15,37]. The steady-state baseline [Ca^2+^]_i_ has been shown to be between pCa 6.4 and 6.6 in rat incisor odontoblasts [38,39]. Thus, consistent with previous reports, our data suggest that the role of PMCA may be to maintain [Ca^2+^]_i_ below 100 nM in odontoblasts under resting conditions. 

The application of the non-selective PMCA inhibitors CE or caloxin 1b1 attenuated the decay of [Ca^2+^]_i_ after hypotonic or high-pH stimulation-induced [Ca^2+^]_i_ increases in both rat and human odontoblasts, showing that PMCA extrudes intracellular Ca^2+^ increased by membrane stretch or high-pH stimuli. Thus, PMCA participates in the maintenance of low [Ca^2+^]_i_ not only during rest but also after intracellular signaling events by stimuli to the dentin surface. Hypotonic and direct mechanical stimuli (which mimic membrane stretch and cell deformation of odontoblasts via dentinal fluid movement in response to multiple external stimuli to the dentin surface) activate Ca^2+^ influx via mechanosensitive ion channels, including TRP vanilloid (TRPV), TRP ankylin 1 (TRPA1), and Piezo1 channels, increasing [Ca^2+^]_i_ in rat and mouse odontoblasts [3,5,29]. The application of high-pH dental materials such as calcium hydroxide and mineral trioxide aggregates on the dentin surface increases the pH of the extracellular environment [40,41], which is detected by odontoblasts in rats and humans [6]. The high-pH environment activates TRPA1 channels and alkali-sensitive metabotropic receptors and stimulates Ca^2+^ release-activated Ca^2+^ channels that induce store-operated Ca^2+^ entry in odontoblasts [6,7]. We previously showed that the functional coupling between Ca^2+^ influx via high-pH- and mechanosensitive-TRP channels and NCX plays an important role in regulating [Ca^2+^]_i_ homeostasis and driving cellular functions in rat, mouse, and human odontoblasts, including the induction of reactionary dentin formation [1,2,3,6,9]. Thus, both NCX and PMCA likely play important roles in reactionary dentin formation by exporting Ca^2+^ to the mineralizing front after the external stimulation of the dentin surface or during the application of high-pH dental materials.

In the mineralization assay with Alizarin red and von Kossa staining, the PMCA inhibitors CE and caloxin 1b1 significantly decreased the mineralization levels. Mineralization in odontoblasts cultured in mineralization medium without PMCA inhibitor (as a control experiment) mimicked the physiological or developmental conditions of dentinogenesis. Thus, these results also demonstrate that Ca^2+^ extrusion via PMCA is essential for dentin mineralization under physiological conditions. High pH, but not hypotonicity, significantly augmented DMP-1 and DSPP mRNA levels. Together with our previous results showing that high-pH stimulation increased mineralization levels via TRPA1 activation in HOB cells [6], it is concluded that extracellular alkaline conditions effectively promote dentinogenesis by increasing not only Ca deposition through the Ca^2+^ extrusion pathway, but also the secretion of non-collagenous extracellular matrix proteins to the mineralizing front. During hypotonic stimulation, Ca^2+^ extrusion following an increase in [Ca^2+^]_i_ was driven by PMCA activity; however, we did not observe any significant differences in levels of DMP-1 and DSPP mRNA under hypotonic conditions as compared to under isotonic conditions. Interestingly, PMCA inhibitors did not affect their mRNA levels in the normal-/high-pH and isotonic/hypotonic extracellular conditions, whereas PMCA activity mediated mineralization under physiological conditions by odontoblasts. Although further studies are needed, these results indicate that Ca^2+^ extrusion processes via PMCA and the secretion of extracellular matrix proteins are regulated by independent signaling pathways. 

In conclusion, this study demonstrates that PMCA contributes to the maintenance of [Ca^2+^]_i_ homeostasis in odontoblasts. PMCA was found to play critical roles not only in physiological dentin formation but also in the pathological tertiary (reactionary) dentin formation induced by multiple external stimuli applied to the dentin surface, as well as by the application of high-pH dental materials on the dentin surface. It has been reported that the activation of neural activity in the brain causes a rapid rise in extracellular pH by removing H^+^ from the external space. This has been proposed to arise from neuronal PMCA activity, which exchanges internal Ca^2+^ for external H^+^ [42]. Therefore, there is considerable interest in understanding whether PMCA activation leads to increased extracellular alkalinity in odontoblasts. Although further studies are needed to clarify the Ca^2+^-H^+^ exchanging ability of PMCA in odontoblasts, compounds that alter PMCA activity may be good candidates for inclusion in dental materials to promote dentinogenesis.

## Figures and Tables

**Table 1:** Primer sets for real-time RT-PCR: plasma membrane Ca^2+^ ATPase (PMCA) and odontoblast marker proteins.

Name	5′-Sequence-3′	GenBank Number
β-actin	Forward TGGCACCCAGCACAATGAA Reverse CTAAGTCATAGTCCGCCTAGAAGCA	NM_001101.5
PMCA1	Forward ACCATATGCTAGAATGCCCACCTC Reverse CTGGTGAAATCTGGGCCCTAAC	NM_001001323.2
PMCA2	Forward AGAGCTTCCGCATGTACAGCAA Reverse CAAGCCATGGGCTCAATCAC	NM_001001331.4
PMCA3	Forward CGTAACGTCTATGACAGCATCTCCA Reverse TCCATGATCAAGTTCACCCACAA	NM_001001344.2
PMCA4	Forward TGGCATGGTTAAATCTGAATGG Reverse CTGCTTCAATTGTAAGGCAAAGG	NM_001001396.2
DMP-1	Forward TCCAGTCTCACAGCAGCTCA Reverse TCTCCGTGGAGTTGCTATCTTC	NM_004407.4
DSPP	Forward TGATAGCAGTGACAGCACATCTGAC Reverse GTTGTTACCGTTACCAGACTTGCTC	NM_014208.3

The conditions for real-time RT-PCR were as follows: 1 cycle at 42 °C for 5 min, followed by 1 cycle at 95 °C for 10 s, 40 cycles at 95 °C for 5 s, and then 60 °C for 30 s. The conditions for dissociation curve analysis were as follows: 1 cycle at 95 °C for 15 s, 1 cycle at 60 °C for 30 s, and 1 cycle at 95 °C for 15 s.

## Data Availability

All data is contained within the article.
